# Design and Crushing Behaviors Investigations of Novel High-Performance Bi-Tubular Tubes with Mixed Multicellular Configurations

**DOI:** 10.3390/biomimetics10090575

**Published:** 2025-09-01

**Authors:** Zhaoji Li, Zhiwen Wang, Dejian Ma, Qingliang Zeng, Dong Ruan

**Affiliations:** 1 College of Mechanical and Electronic Engineering, Shandong University of Science and Technology, Qingdao 266590, China; lzj0809zxw@sdust.edu.cn (Z.L.); dejian_ma@sdust.edu.cn (D.M.); 2 College of Transportation, Shandong University of Science and Technology, Qingdao 266590, China; zwwang2018@sdust.edu.cn; 3 College of Safety and Environmental Engineering, Shandong University of Science and Technology, Qingdao 266590, China; 4 Shandong Key Laboratory of Collaborative Mining Technology for Intelligent Mine Equipment, Qingdao 266590, China; 5 Department of Mechanical and Product Design Engineering, School of Engineering, Swinburne University of Technology, Melbourne, VIC 3122, Australia

**Keywords:** non-dimensional parameter, crushing behaviors, bi-tubular tubes, multicellular configurations, high-performance tubes

## Abstract

Thin-walled structures have been extensively adopted as energy absorbers in various engineering fields. The energy accumulated in the coal and rock is released instantly, resulting in varying degrees of damage and failure to support equipment. To improve the crushing performance of underground support equipment, a metal thin-walled tube with high-bearing capacities is placed in the column as an energy-absorbing column. Based on the characteristics of non-dimensional parameters governing the crashworthiness of thin-walled tubes by the author’s team, a type of high-performance bi-tubular tube (HPBT) with mixed multicellular configurations is innovatively proposed. First, the finite element models of the HPBTs are established in LS-DYNA, and the accuracy of the FE model is verified by crushing tests. Second, the theoretical model of the mean crushing force (*MCF*) is derived. Moreover, the effects of the cross-sectional shapes and the wall thickness gradient distribution on the deformation modes and crashworthiness are investigated. The results show that the design strategies of the bi-tubular structures mixed multicellular configurations significantly improve the values of *ω*. The *MCF* of HPBT_C2 is 4458.0 kN, which is 28% and 56% higher than those of the conventional circular tube and square tube. The theoretical *MCF* is consistent with the simulated *MCF*, with a maximum discrepancy of 6.0%. The gradient distribution (*k*) of wall thickness significantly affects the crushing behaviors of the HPBT. Considering the energy absorption efficiency, the crushing stability, and the wall thickness gradient distribution, the HPBT_C2 with *k* = 0.6 has the best overall performance. The results can provide insights and guidelines for designing energy absorption devices with superior crashworthiness for support equipment.

## 1. Introduction

**Figure 1:**
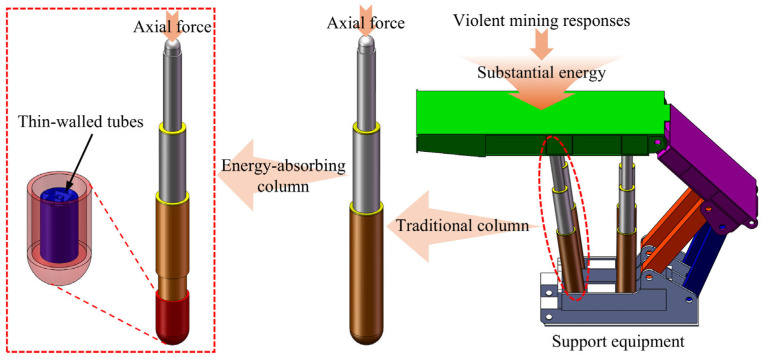
Schematic diagram of the support equipment and energy-absorbing columns.

As coal mining operations progress to greater depths, violent mining responses are inevitable [1,2,3]. The fracture and sliding of rock strata cause instantaneous impacts on the support equipment, resulting in varying degrees of damage and failure [4,5], as shown in Figure 1. This presents a significant risk to the safety and lives of workers because the substantial energy generated by the movement of rock strata cannot be dissipated promptly.

To improve the crushing performance of the support equipment, researchers have continuously improved the strength of the columns by improving the material strength and increasing the volume of the cylinder. However, this puts higher requirements on the hydraulic components. In this context, more and more scholars use the energy absorption of thin-walled structures to improve the crushing performance of support equipment [6,7,8,9]. Installing thin-walled structures on the columns or canopies significantly improves the crashworthiness of the support equipment.

Based on this, a metal thin-walled tube is placed in the column as an energy-absorbing column, as shown in Figure 1. A pressure head is welded at the bottom of the one-level cylinder, which ensures that the thin-walled tubes undergo axial deformation under the impact load. Using its energy absorption, when the loading capacity of thin-walled tubes exceeds the initial peak crushing force (*IPCF*), thin-walled tubes deform plastically and produce plastic folding to dissipate the kinetic energy and protect the equipment and workers.

The *IPCF*, referring to the critical load at which the thin-walled tubes begin to undergo plastic deformation, is the preferred parameter to evaluate the high-bearing capacity of the columns. The *IPCF* is expected to be as follows:

(1) $$F_{C1}<IPCF<F_{C2},$$

where *F_C_*_1_ is the working resistance of the column, set to 5500 kN. *F_C_*_2_ is the critical failure load of the column, which is usually 1.2–1.5 times the working resistance of the column (5500–8250 kN).

Thin-walled structures play an important role in various engineering fields due to their easy processing, light weight, and high energy absorption efficiency [10,11,12,13,14]. For a long time, researchers have conducted extensive investigations on the energy absorption characteristics of thin-walled structures, especially thin-walled circular and square tubes. Alexander [15] first established a theoretical model for the thin-walled circular tubes during axial crushing, which laid the theoretical foundation for the development of thin-walled structures. Later, Wierzbicki et al. [16] explored the deformation modes of thin-walled square tubes and proposed the super folding element to predict their mean crushing force. Fan et al. [17] numerically and experimentally investigated the crashworthiness of thin-walled tubes with hexagonal, octagonal, dodecagonal, and hexadecagonal cross-sections. The results showed that the energy absorption of single-celled tubes is closely related to their cross-sectional shapes. However, the energy absorption of single-celled tubes is relatively limited, and the tubes are prone to Euler buckling. To this end, researchers have gradually begun to look for novel thin-walled tubes.

In recent years, multi-celled tubes have attracted great attention from scholars [18,19]. Their excellent energy absorption characteristics and load-bearing capacities have good application prospects in the field of buffering [20,21,22,23]. Chen et al. [24] proposed a simplified super folding element (SSFE) theory and derived the folding wavelength and mean crushing force of multi-celled square tubes. Nagarjun et al. [25] compared the crushing behaviors of multi-celled and single-celled tubes through numerical simulations and crushing tests. The results showed that adding rib plates significantly enhances the crashworthiness of thin-walled tubes. Zhang et al. [26] proposed a novel thin-walled tube with various graded thickness and reported the effect of gradient types on crashworthy performance.

Although these cited studies on multi-celled tubes have revealed significant energy absorption, the potential for lightweight and higher energy absorption efficiency remains unrealized. This is imminent. In recent years, bionics have been successfully applied to various engineering fields [14,27,28,29,30]. The biological structures obtained after a long period of survival of the fittest often have excellent mechanical properties and important structural imitation values. Using the concept of bionics to learn from biological structures is an effective way to design and develop novel thin-walled tubes. Zhang et al. [31] designed a group of multi-cell elliptic tubes (METs) imitating the wing structure of the dragonfly and reported that METs have better crashworthy performance in oblique crushing. By mimicking the microstructure of the beetle, Zhang et al. [32] and Deng et al. [33] proposed a strategy to fill circular tubes at different multi-cell tubes positions, respectively. Ha et al. [34] explored the crashworthiness of bionic bi-tubular tubes imitating the structure of giant lily water venation. Combining the concepts of hierarchy and bionics, Zhang et al. [35] and Tsang et al. [36] proposed hierarchical tubes inspired by the structures of spider webs and tendons, respectively. Cai and Deng [37] explored a fractal hierarchical honeycomb inspired by the branching structure of a tree and discussed the effects of wall thickness, bifurcation factor, and bifurcation angle.

The above research examines the adoption of various design strategies, such as multicellular, filled, bionic, and hierarchical strategies, to improve the crashworthiness of thin-walled tubes. However, the design strategies are not quantified. Our previous research [38] proposed a single non-dimensional parameter to design high-performance thin-walled tubes. In addition, compared with other engineering fields, underground support equipment requires high-bearing capacity, which cannot be met by conventional thin-walled tubes within the limited geometrical parameters. Therefore, to improve the crushing performance of the underground support equipment, considering the high values of *ω* (with a large *R_T_* and a small *R_G_*), low-cost manufacturing, and stable deformation modes, a new type of high-performance bi-tubular tube (HPBT) with mixed multicellular configurations is proposed. The finite element models of the HPBTs are established in LS-DYNA, and the accuracy of the finite element models is verified by crushing tests. The theoretical model of the mean crushing force is derived. Moreover, the deformation modes and crashworthiness of the HPBT with different topological configurations are investigated, and the effect of the wall thickness gradient distribution is discussed. Finally, combined with the crashworthiness indicators, the HPBT with the optimal cross-sectional shapes and geometrical parameters is determined. The results provide practical significance and theoretical guidance for the design of energy absorption devices for underground support equipment.

## 2. Design and Methodology

### 2.1. Structural Geometry

Based on our previous research [38], the load-bearing capacity and energy absorption efficiency of thin-walled tubes increase monotonically with a non-dimensional governing parameter, *ω*, which is determined by $\sqrt{R_{T}}/R_{G}$, where *R_T_* is the coefficient used to calculate the energy dissipation by the junction elements in the cross-section of the tube, and *R_G_* is ratio of the total side length of a tube to the total length of a reference tube with the same apparent cross-sectional area and mass. Conventional single-celled tubes and multi-celled tubes with low values of *ω* cannot meet the high bearing requirements for underground support equipment. Furthermore, although hierarchical tubes, honeycomb structures, and metamaterials have high values of *ω*, the fabrication of their complex cross-sectional shapes is expensive, the installation space is limited, and the lobes generated during the crushing process are mainly concentrated in the mixed modes. Therefore, efforts have been made to develop the cross-sectional topology of tubes to increase the value of *ω*, thereby enhancing their energy absorption. Tubes with a large *R_T_* and a small *R_G_* will lead to a large *ω*, resulting in improved crushing force and energy absorption. To increase *R_T_*, more junction elements are expected to be introduced. Conversely, to reduce *R_G_*, these junction elements are expected to be strategically placed in the central region of the tube. Accordingly, in this study, a multi-celled configuration is employed in the central region, with ribs connecting it to the outer tube to enhance structural performance.

**Figure 2:**
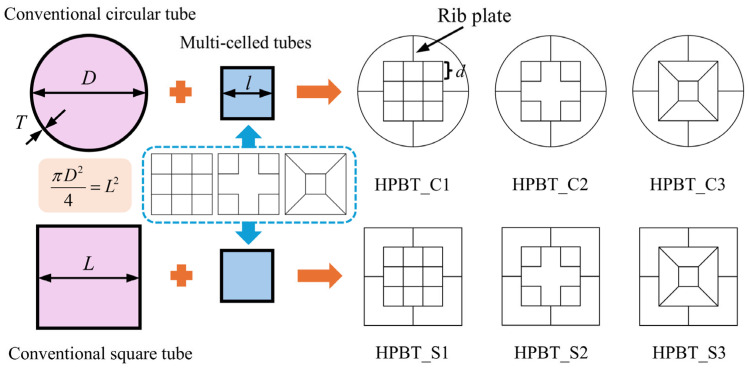
Configuration evolution and cross-sectional shapes of high-performance bi-tubular tubes proposed.

In this paper, a high-performance bi-tubular tube (HPBT) with mixed multicellular configurations is proposed and investigated. The cross-sectional shapes are shown in Figure 2. Each tube has three parts: the outer single-celled tubes, the middle rib plates, and the inner multi-celled tubes. Among them, the outer single-celled tubes are conventional circular tubes (CCT) and conventional square tubes (CST), respectively. For the convenience of description, the two types of high-performance bi-tubular tubes proposed are named “HPBT_C*n*” and “HPBT_S*n*”, respectively. According to the different multicellular configurations of the inner tubes, *n* is 1, 2, and 3, respectively.

To facilitate a fair comparison of the high-performance bi-tubular tubes proposed, the apparent areas enclosed by the outmost sides of the outer tubes are the same. Moreover, all the tubes proposed have the same wall material (Q690), the same height (480 mm), and the same mass (37.84 kg). The geometrical parameters of the high-performance bi-tubular tubes are listed in Table 1.

### 2.2. Experimental Tests

**Figure 3:**
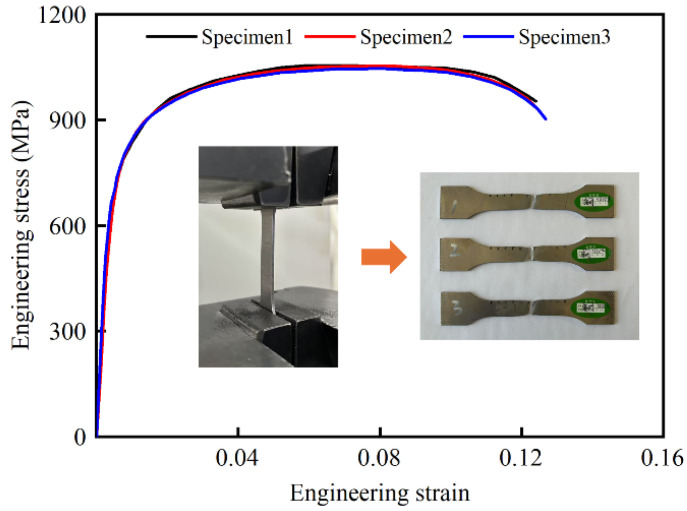
The engineering stress–strain curves of the three specimens.

To match the load-bearing capacity requirements of the columns, Q690 was selected as the material of high-performance bi-tubular tubes. The material properties were obtained from the tensile tests conducted on three dog-bone coupons using LD26.305 electronic universal testing machine (Labsans Testing Machine Co. Ltd., Shenzhen, China). The engineering stress–strain curves of three tensile tests are shown in Figure 3. Evidently, the fractures and stress–strain curves of the coupons show good consistency. The average mechanical properties of the material are listed in Table 2.

**Figure 4:**
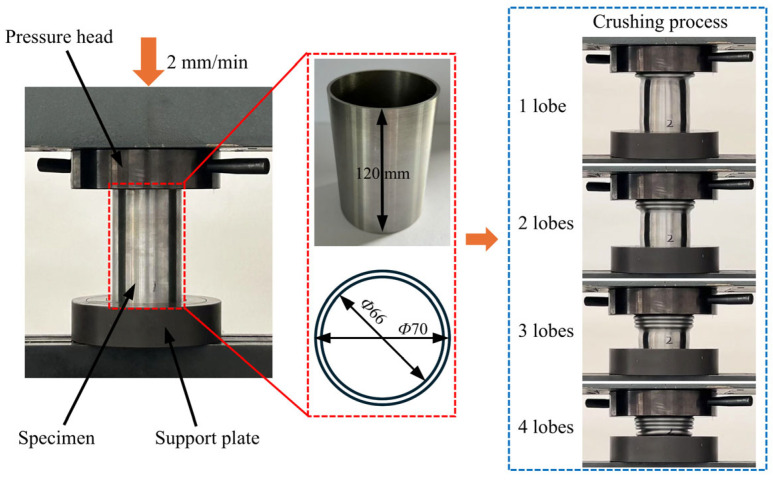
Quasi-static crushing tests and crushing process.

**Figure 5:**
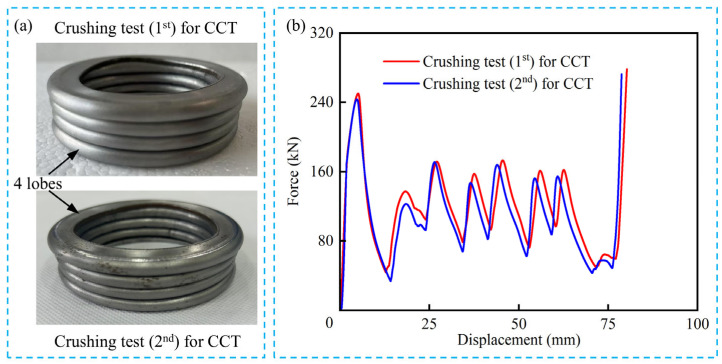
Quasi-static crushing test results: (**a**) the deformation modes of two CCT specimens; (**b**) the force–displacement curves of two CCT specimens.

Two CCT tubes were experimentally tested using LD26.305 electronic universal testing machine (Labsans Testing Machine Co. Ltd., Shenzhen, China). Due to the limitation of the crushing force for the universal testing machine, the height, outermost diameter, and wall thickness of the CCT specimens were 120 mm, 70 mm, and 2 mm, respectively. The diameter-to-thickness ratio of the CCT specimens is 35, and the length-to-diameter ratio is 1.7, which is prone to progressive deformation modes. As shown in Figure 4, a CCT tube was placed between the pressure head and the support plate. Subsequently, the pressure head moved downward along the axial direction at a speed of 2 mm/min, with a crushing distance of 100 mm. The embedded sensors of the LD26.305 were utilized to record the crushing force and distance. It is evident in Figure 4 and Figure 5a that the deformation mode of CCT tubes is progressive with four uniform lobes generated. Moreover, the force–displacement curves of CCT specimens (Figure 5b) are regular and stable. Both the deformation modes and the force–displacement curves demonstrate strong consistency between the two specimens.

### 2.3. Crashworthiness Indicators

Due to the different geometrical parameters and cross-sectional shapes, the crushing behaviors of high-performance bi-tubular tubes are different, which affects their energy absorption characteristics. To select reliable energy absorbers, seven indicators are employed to evaluate the energy absorption characteristics, namely, energy absorption (*EA*), effective stroke ratio (*ESR*), specific energy absorption (*SEA*), mean crushing force (*MCF*), effectiveness of energy absorption (*EEA*), crushing force efficiency (*CFE*), and undulation of load-carrying capacity (*ULC*) [39,40].

(1) The *EA* refers to the total energy absorbed within the effective stroke during the crushing and is defined as follows:

(2) $$EA=\int_{0}^{\delta }F(z)dz,$$

where *F*(*z*) is the instantaneous axial crushing force in kN, and *δ* is the effective stroke in mm.

(2) The *ESR* represents the utilization ratio of materials and is defined as follows:

(3) $$ESR=\frac{\delta }{h},$$

where *h* is the height of the tubes in mm.

(3) The *SEA* refers to the energy absorbed by unit mass, and it denotes how effectively the tubes absorb energy and is defined as follows:

(4) $$SEA=\frac{EA}{m},$$

where *m* denotes the mass of the tubes in kg.

(4) The effective stroke divided by the total energy absorbed is known as the *MCF*, which reflects the load-bearing capacity of the tubes during the crushing process. The *MCF* can be calculated as follows:

(5) $$MCF=\frac{EA}{\delta },$$

(5) *EEA* is a non-dimensional indicator that represents the efficiency of the energy absorption of tubes and is determined as follows:

(6) $$EEA=\frac{EA}{VY}=\frac{EA}{hAY},$$

(6) The *CFE* refers to the stability of plastic deformation during the crushing process and can be expressed as follows:

(7) $$CFE=\frac{MCF}{IPCF}\times 100\%,$$

(7) The *ULC* refers to the fluctuation of the crushing force during the folding process. The larger it is, the more violent the fluctuation of the crushing force is. It can be calculated as follows:

(8) $$ULC=\frac{\int_{0}^{\delta }\left|F(z)-MCF\right|dz}{\int_{0}^{\delta }F(z)dz},$$

### 2.4. Finite Element Models

**Figure 6:**
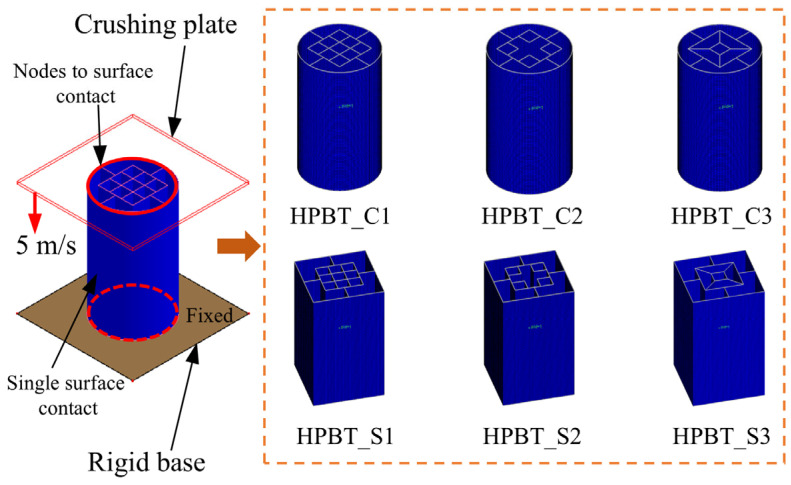
Finite element models.

Based on the cross-sectional topologies of the high-performance bi-tubular tubes (Figure 2), finite element models are established using LS-DYNA (4.8.17), as shown in Figure 6. Each finite element model consists of three parts, namely, the crushing plate, the high-performance bi-tubular tube, and the rigid base. Among them, the crushing plate and the rigid base are both rigid bodies and do not deform. The high-performance bi-tubular tubes are modeled using shell elements, with five integration points defined through the thickness direction.

**Figure 7:**
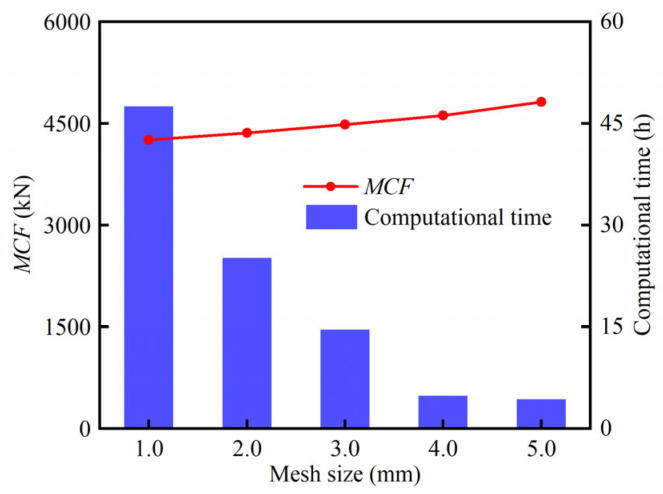
Mesh convergence analysis.

To determine the appropriate mesh size, a convergence analysis was carried out. HPBT_C1 was selected, and its mesh sizes were 1.0 mm, 2.0 mm, 3.0 mm, 4.0 mm, and 5.0 mm, respectively. The mean crushing force (*MCF*) and computational time are both affected by the mesh size, as shown in Figure 7. Moreover, the discrepancies of 2.4% and 5.4% are obtained in the *MCF* between 2.0 mm and 1.0 mm and between 3.0 mm and 2.0 mm, respectively. Considering the balance of the computational time and efficiency, a mesh size of 2 mm was selected for all the tubes proposed. In addition, the material of high-performance bi-tubular tubes is Q690, whose engineering stress–strain curve is shown in Figure 3. In this study, the strain rate effect was not considered in the FE models [6,41].

All the tubes studied are placed between the crushing plate and the rigid base. The crushing plate is set to a crushing speed of 5 m/s to simulate the impact load, and the rigid base is fully constrained. Based on our previous research [42], the automatic nodes-to-surface contact is set between tubes and plates. To avoid self-penetration of the tube walls, the thin-walled tube itself is set to automatic single-surface contact. The friction coefficient in both types of contacts is set to 0.15 [43,44].

### 2.5. Experimental Validation

**Figure 8:**
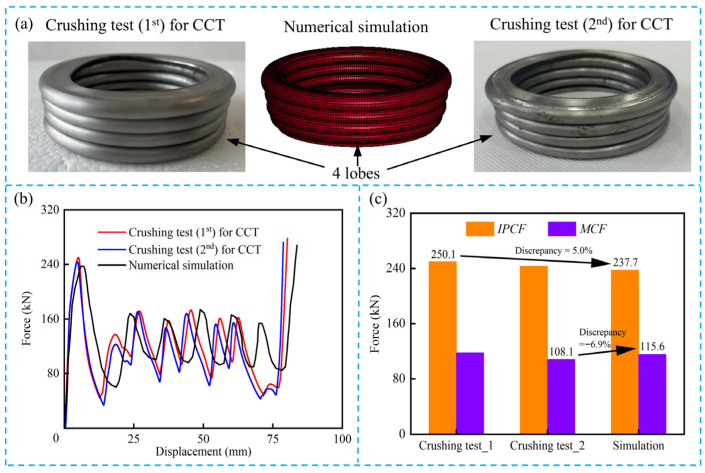
Comparison of experimental and numerical results: (**a**) the deformation modes of CCT in both experiment and simulation; (**b**) the force–displacement curves of CCT in both experiment and simulation; (**c**) the experimentally measured and numerically simulated discrepancies of *IPCF* and *MCF*.

To verify the accuracy of the finite element models, quasi-static crushing tests have been carried out on the conventional circular tubes in Section 2.2. Based on the structural dimensions of the specimen in Figure 4, the finite element model of CCT was established, and its height, outermost diameter, and wall thickness were 120 mm, 70 mm, and 2 mm, respectively. The finite element model was constructed using the same method described in Section 2.4. As shown in Figure 8a, the deformation modes of CCT in the crushing test and the numerical simulation are almost consistent. Four lobes are formed during the crushing process. Moreover, Figure 8b shows the force–displacement curves of CCT obtained from experimental tests and simulations. The fluctuations of the crushing forces are similar. From Figure 8c, the maximum discrepancies in *IPCF* and *MCF* between the experiment and simulation are 5.0% and −6.9%, respectively. Therefore, the accuracy of the finite element models is fully verified and can be used for subsequent simulations.

## 3. Theoretical Analysis

To quantitatively investigate the relationship between the topological configurations of the high-performance bi-tubular tubes and energy absorption, a theoretical model was established. Based on the simplified super folding element (SSFE) theory [24], it is assumed that the folding deformation of tubes during the crushing process is periodic, and the wavelength of each folding element is the same [22,38,43,45]. A folding element is composed of three horizontal plastic hinge lines. For each folding element, the energy balance relationship under the external force can be expressed as follows:

(9) $$2H\cdot MCF\cdot \eta =E_{b}+E_{m},$$

where 2*H* is the folding wavelength, mm; *η* is the coefficient of effective stroke, which is equal to *ESR*; and *E_b_* and *E_m_* refer to the bending energy and the membrane, respectively.

### 3.1. Bending Energy

**Figure 9:**
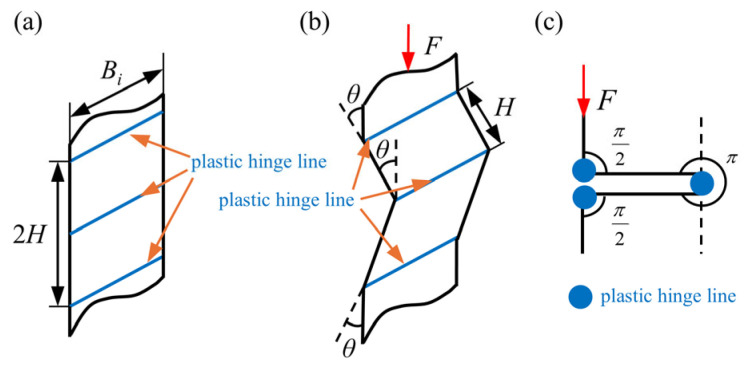
The bending deformation of plastic hinge lines during each folding process: (**a**) before crushing; (**b**) bending deformation with the rotation angle θ. (**c**) In the fully folded state, the rotation angles of the three plastic hinge lines are π/2, π, and π/2, respectively.

The bending energy of each folding process is equal to the sum of the energy dissipated by the three plastic hinge lines, as shown in Figure 9. Under the ideal conditions, the rotation angles of three horizontal plastic hinge lines are π/2, π and π/2, respectively. Therefore, the bending deformation energy can be defined as follows [46]:

(10) $$E_{b}=\sum_{i}^{3}\theta_{i}B_{i}M=2\pi MB,$$

where *θ_i_* is the rotation angle of the horizontal plastic hinge line; *B_i_* the side length (in mm) of the folding element; *M* is the plastic moment per unit length (in N); and *B* is the total length (in mm) of the cross-section of the high-performance bi-tubular tube.

Then, according to the material properties and wall thickness, the plastic moment can be defined as follows:

(11) $$M=\frac{1}{4}\sigma_{0}T^{2},$$

where *σ*_0_ is the flow stress (in MPa) of the material, which is supposed to be equal to the average of the yield stress and ultimate stress of tube wall material [38].

### 3.2. Membrane Energy

**Figure 10:**
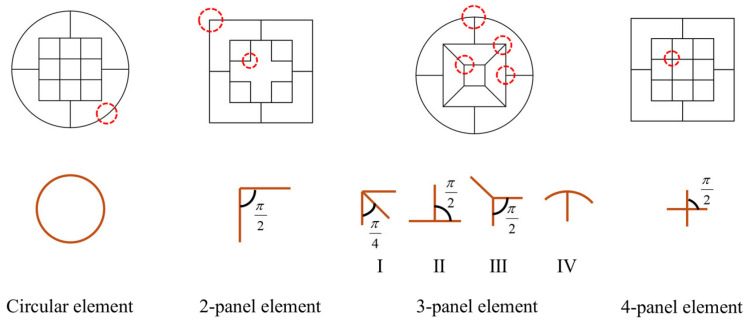
Schematic diagram of junction elements of all the tubes proposed.

The membrane energy is determined by the plastic deformation of different junction elements. As shown in Figure 2, the types and numbers of junction elements of various high-performance bi-tubular tubes are different. The six types of the proposed tubes consist of four types of junction elements, namely, the circular element, the two-panel element, the three-panel element with four forms (i.e., I, II, III, and IV), and the four-panel element, as shown in Figure 10.

**Figure 11:**
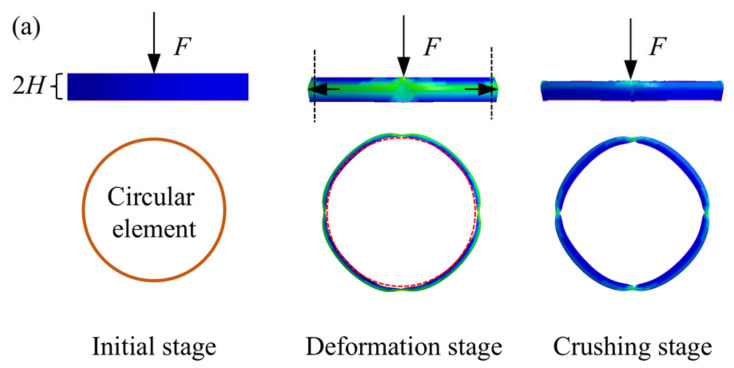

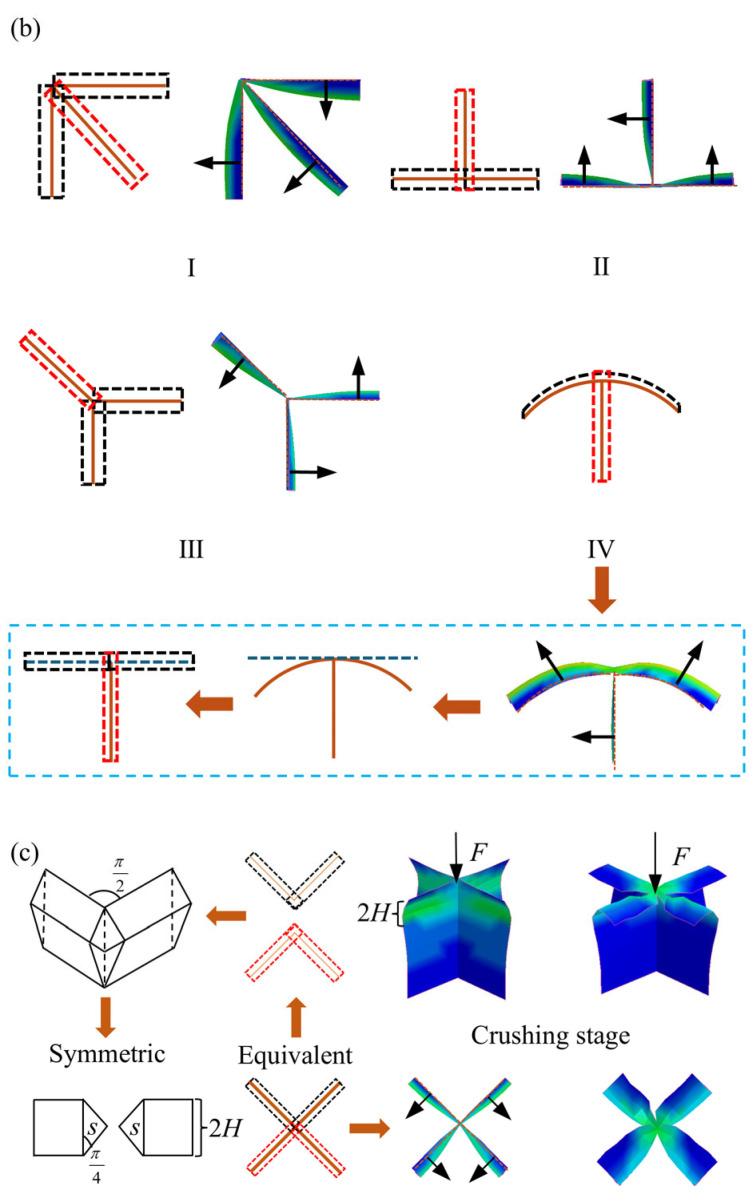
Deformation characteristics of junction elements in folding wavelength: (**a**) circular elements; (**b**) three-panel elements with four forms (i.e., I, II, III, and IV); (**c**) four-panel elements.

For the membrane energy of different junction elements, the deformation area is integrated and then accumulated to obtain the membrane energy of tubes. To this end, the deformation characteristics of the junction elements during the crushing process are plotted in Figure 11.

### 3.3. Mean Crushing Force

From the design strategies of high-performance bi-tubular tubes in Section 2.1, all the tubes proposed follow the same four principles. Therefore, according to the definitions of *R_G_*, *R_T_*, and *ω* in our previous research [38,42], the mean crushing force of tubes can be expressed as follows:

(12) $$MCF=\frac{\sqrt{2\pi }}{4\eta }k_{d}\sigma_{0}B_{re}^{0.5}T_{re}^{1.5}\omega ,$$

where *k_d_* is a dynamic coefficient. *B_re_* and *T_re_* represent the total length and wall thickness of the reference tube, respectively. The reference tube selected here is CC; that is, *B_re_* is 879.6 mm, and *T_re_* is 11.46 mm. *ω* is a non-dimensional parameter that reflects the crashworthiness of the tubes and can be expressed as $\omega =\sqrt{R_{T}}/R_{G}$. Among them, *R_T_* is the coefficient to calculate the energy dissipation of the tubes and is closely related to the types and numbers of junction elements in the cross-section. *R_G_* refers to the ratio of the total length of the other tubes to that of the reference tube (CCT).

The membrane energy of various junction elements can be calculated by our previous research [38,42]. Combining Equation (12) and Table 1, the *R_G_*, *R_T_*, *ω*, and theoretical *MCF* are listed in Table 3. Evidently, the design strategies of the bi-tubular structures mixed multicellular configurations significantly improve the *ω* and *MCF* of tubes. Since the *MCF* increases with the increase in ω, HPBT_C2 has the largest *ω*, and also, it has the highest *MCF*, which is 4681.1 kN.

## 4. Numerical Results

### 4.1. Deformation Modes

**Figure 12:**
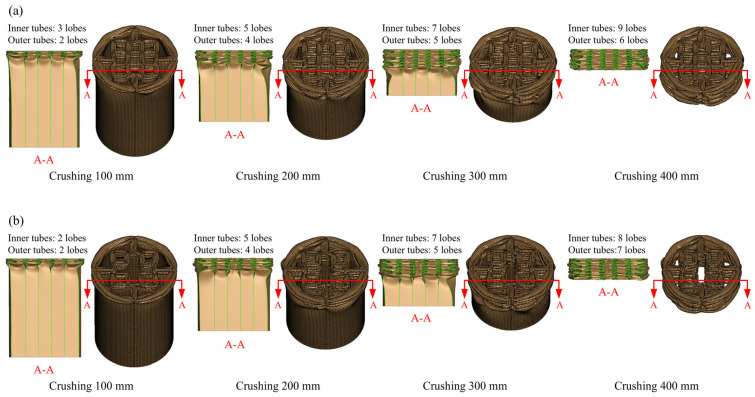

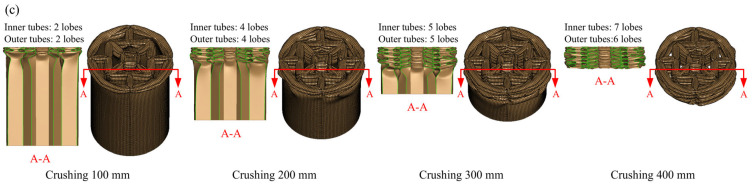
Deformation modes of the HPBT with conventional circular tubes as the outer single-celled tube: (**a**) HPBT-C1; (**b**) HPBT-C2; (**c**) HPBT-C3.

**Figure 13:**
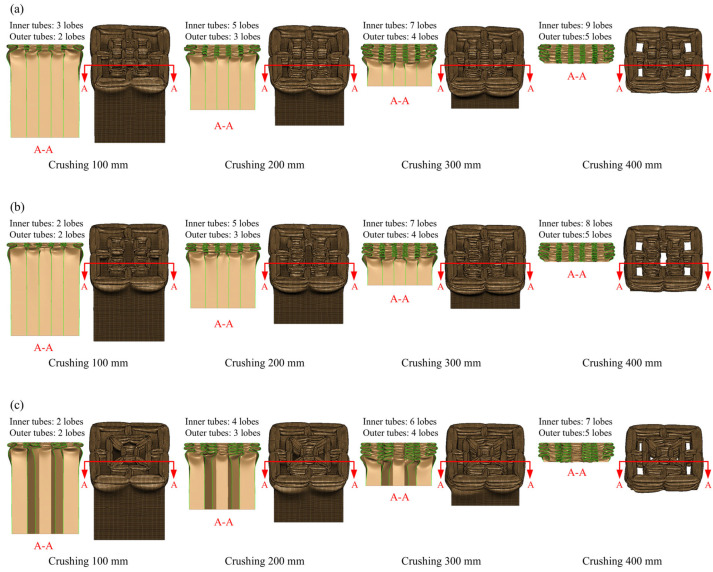
Deformation modes of the HPBT with conventional square tubes as the outer single-celled tube: (**a**) HPBT-S1; (**b**) HPBT-S2; (**c**) HPBT-S3.

The deformation modes of HPBT_C*n* and HPBT_S*n* are displayed in Figure 12 and Figure 13, respectively. Clearly, the inner tubes of HPBT_C*n* and HPBT_S*n* exhibit similar deformation modes. When the inner multicellular configurations are identical, both demonstrating stable folding deformation (Figure 12). Moreover, with the increase in the number of cells in the inner tubes, more lobes are generated during the crushing process. This is because increasing the number of cells reduces the side length, resulting in smaller folding wavelengths and, consequently, a greater number of lobes. In addition, the number of lobes in the inner tubes is more than those of the outer single-celled tubes due to smaller side length.

As shown in Figure 12, the outer circular tube presents mixed deformation modes during the crushing process. In the early stage of crushing, progressive (i.e., concertina) deformation modes are generated; then, diamond deformation modes gradually appear. This is because the diameter-to-thickness ratio of the outer circular tube is 56 and the length-to-diameter ratio is 1.7, which is prone to mixed deformation modes [47]. Moreover, the outer tubes of HPBT_C*n* have more lobes than HPBT_S*n* (Figure 13).

Differently, as shown in Figure 13, the HPBT_S*n* exhibits progressive folding deformation and stable plastic stretching, which is conducive to reducing the fluctuation of the crushing force. To guide the thin-walled tubes to produce symmetric deformation, the right-angle corners can be appropriately increased.

### 4.2. Force–Displacement Curves

**Figure 14:**
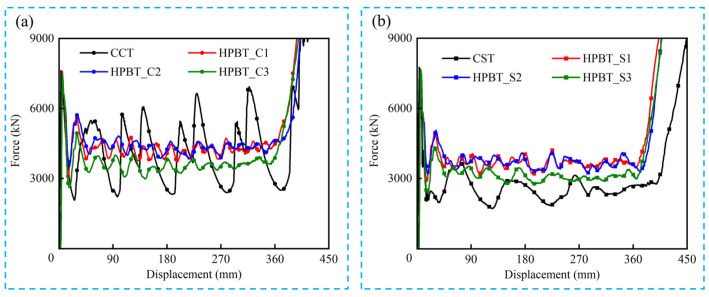
Force–displacement curves of all the tubes studied: (**a**) CCT and HPBT_C*n*; (**b**) CST and HPBT_S*n*.

The force–displacement curves of HPBT_C*n* and HPBT_S*n* are displayed in Figure 14. Each force–crush displacement curve of the high-performance bi-tubular tubes with various cross-sectional shapes has three distinct stages, namely, the elastic stage, the plateau stage, and the densification stage. In the elastic stage, the crushing force increases sharply within an extremely short distance. Until the crushing force reaches the first peak, i.e., *IPCF*, the tubes begin to deform plastically. After that, the crushing force gradually stabilizes near the *MCF* and shows a sawtooth fluctuation. The tube is in the plateau stage, which is the key stage for energy absorption. Continuous axial crushing eventually leads to close contact between the lobes. The crushing force shows an exponential growth and the tubes are in the densification stage.

As shown in Figure 14, six types of the HPBTs exhibit less violent fluctuations in the platform crushing stage compared with the conventional circular and square tubes. This significantly improves the crushing behaviors of tubes. Moreover, the crushing forces of HPBT_C1 and HPBT_C2 are higher than that of HPBT_C3 from Figure 14a, which indicates that the right-angle corners in the cross-section are conducive to improving the crushing force. Similarly, Figure 14b shows that the crushing forces of HPBT_S1 and HPBT_S2 are higher than that of HPBT_S3. In addition, the plateau crushing force of HPBT_C*n* is higher than that of HPBT_S*n*. This is because increasing the number of lobes reduces the folding wavelengths and, consequently, increases the plateau crushing force (Figure 12 and Figure 13).

### 4.3. Crashworthiness Analysis

The crashworthiness indicators of all the tubes studied are listed in Table 4. The effective stroke of the CST is the largest among all the tubes studied. Moreover, the *ESR* of the tubes decreases with the introduction of the inner multicellular configurations, especially for HPBT_C1 and HPBT_S1, whose *ESR*s are the lowest of their respective types due to the largest number of cells in the inner tubes. The *ESR*s of HPBT_C1 and HPBT_S1 are 11% and 10% lower than that of the CST, respectively.

**Figure 15:**
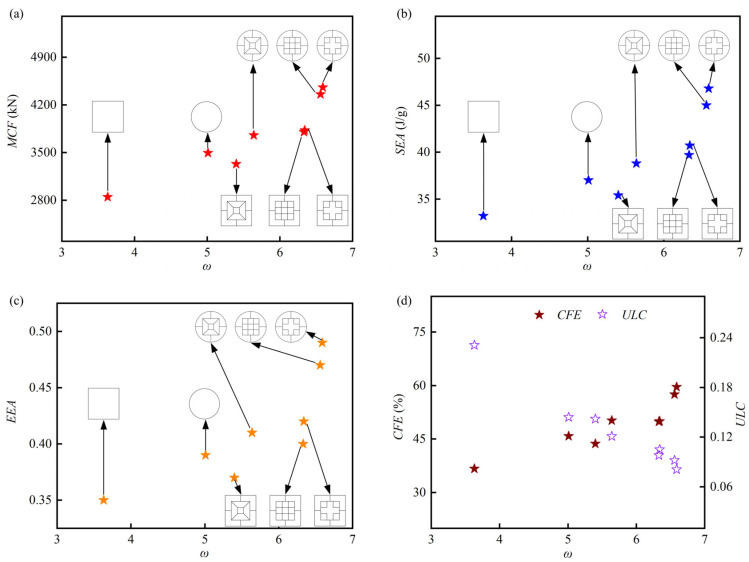
Crashworthiness indicators versus the ω of all the tubes studied: (**a**) *MCF*; (**b**) *SEA*; (**c**) *EEA*; (**d**) *CFE* and *ULC*.

The crashworthiness indicators versus the *ω* of all the tubes studied are shown in Figure 15. The *MCF*, *SEA*, and *EEA* of tubes increase with the increase in ω, except for HPBT_S3. The design strategies of the bi-tubular structures with mixed multicellular configurations effectively increases the *ω*, which enhances the crushing behaviors of the tubes. Specifically, the *MCF*s of HPBT_C2 and HPBT_S2 are 4458.0 kN and 3826.5 kN, which are 28% and 34% higher than the CCT and CST, respectively. Moreover, the crushing behaviors of HPBT_C3 and HPBT_S3 are not significantly improved compared with the conventional single-celled tubes. For example, their *SEA*s are 38.8 J/g and 35.4 J/g, respectively, which are only 7% and 5% higher than those of CCT and CST. This phenomenon can also be observed from their force–displacement curves (Figure 14). The *MCF* and *SEA* of HPBT_C2 are the maximum values among all the tubes studied, which are 4458 kN and 46.8 J/g, respectively. The *EEA* of HPBT_C2 is 0.49, which is 26% and 40% higher than those of the CCT and CST, respectively. From Table 1, it can be seen that the wall thickness of HPBT_C2 is 4.71 mm, which is the maximum value across the six types of high-performance bi-tubular tubes. This is because the effect of the wall thickness accounts for a larger proportion of the overall effect.

Figure 15d displays the *CFE* and *ULC* versus the *ω* of all the tubes studied. Both the indicators reflect the stability of the tubes during the crushing process. An ideal tube has a higher *CFE* and a lower *ULC*. The *CFE* of HPBT_C2 is 59.6%, which is 30% and 62% higher than that of CCT and CST, respectively. This means that the difference between *MCF* and *IPCF* of HPBT_C2 is the smallest, and the fluctuation of the crushing force is the least violent during the platform crushing stage shown in Figure 14. Similarly, the *ULC* of HPBT_C2 is also the lowest value across all the tubes studied (0.081). In summary, the energy absorption efficiency and crushing stability of HPBT_C2 are superior to those of high-performance bi-tubular tubes with other cross-sectional shapes.

Furthermore, the theoretical *MCF*s of all the tubes studied are derived and calculated in Section 3.3. Based on this, the comparison results between the simulated *MCF* and the theoretical *MCF* are listed in Table 5. Obviously, the theoretical value of *MCF* is in good agreement with the simulation value, with a maximum discrepancy of 6.0%, which is within the allowable discrepancy range. Therefore, the theoretical model can accurately predict the energy absorption characteristics of the high-performance bi-tubular tubes.

### 4.4. Effect of Wall Thickness Gradient Distribution

Wall thickness has a significant effect on the energy absorption and load-bearing capacity of the tubes. It can be concluded from Section 4.3 that HPBT_C2 has excellent crashworthiness. Therefore, HPBT_C2 is selected to discuss the effect of wall thickness gradient distributions.

**Figure 16:**
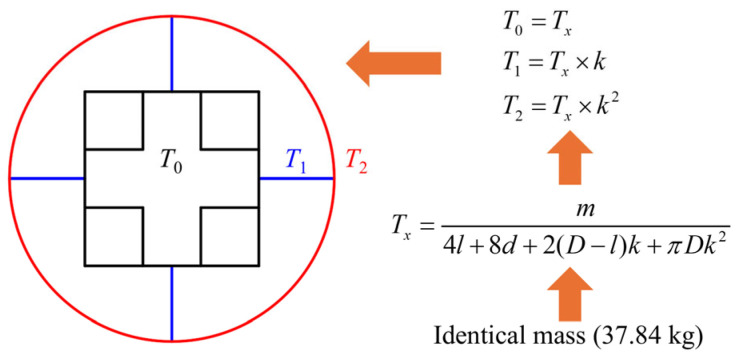
Schematic diagram and calculation of wall thickness gradient distributions of HPBT_C2.

Based on the cross-sectional shapes from Figure 2, each tube has three parts: the outer single-celled tubes, the middle rib plates, and the inner multi-celled tubes. As shown in Figure 16, the wall thicknesses of the inner multi-celled tubes, the middle rib plates, and the outer single-celled tubes are defined as *T*_0_, *T*_1_, and *T*_2_, respectively. Moreover, the coefficients of wall thickness gradient distribution increase step by step from the insider to the outside and are defined as *k*. The values of *k* are set to 0.4, 0.6, 0.8, 1.0, 1.2, and 1.4, respectively. All the HPBT_C2 with different wall thickness gradients have the same wall material (Q690), the same height (480 mm), and the same mass (37.84 kg). And the calculation of the wall thickness of each layer is shown in Figure 16. The detailed data of the wall thickness distribution of the HPBT_C2 are listed in Table 6.

**Figure 17:**
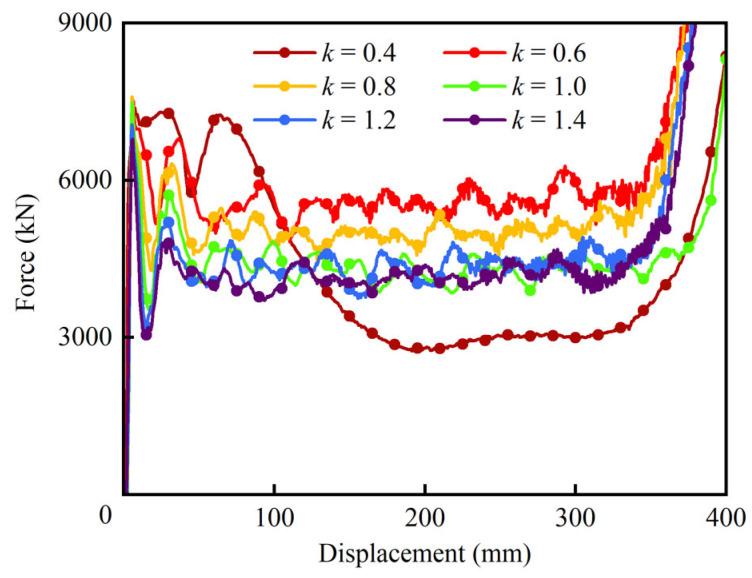
Force–displacement curves of HPBT_C2 with different values of *k*.

The force–displacement curves of HPBT_C2 with different values of *k* are displayed in Figure 17. Clearly, the wall thickness gradient distributions have a significant influence on the crashworthiness of high-performance bi-tubular tubes. At *k* = 0.4, the crushing force of HPBT_C2 fluctuates violently without the plateau stage, making it unsuitable as an energy absorber. However, when *k* increases to 0.6, the crushing performance of HPBT_C2 improves rapidly. And the crushing force of the tubes is the highest when *k* is between 0.4 and 1.4. Moreover, *k* is equal to 1, which means that the wall thickness distribution in the cross-section of HPBT_C2 is uniform, and its crushing force fluctuation is the same as Figure 14a. Furthermore, when *k* is greater than 1, the crushing platform stage of HPBT_C2 decreases, and in particular, the crushing force of HPBT_C2 is the smallest when *k* is 1.4. The main reason for this trend is that the inner multi-celled tubes play a crucial role in the crashworthy performance of the high-performance bi-tubular tubes. Increasing the material in the inner tubes enhances the crashworthiness of the tubes.

**Figure 18:**
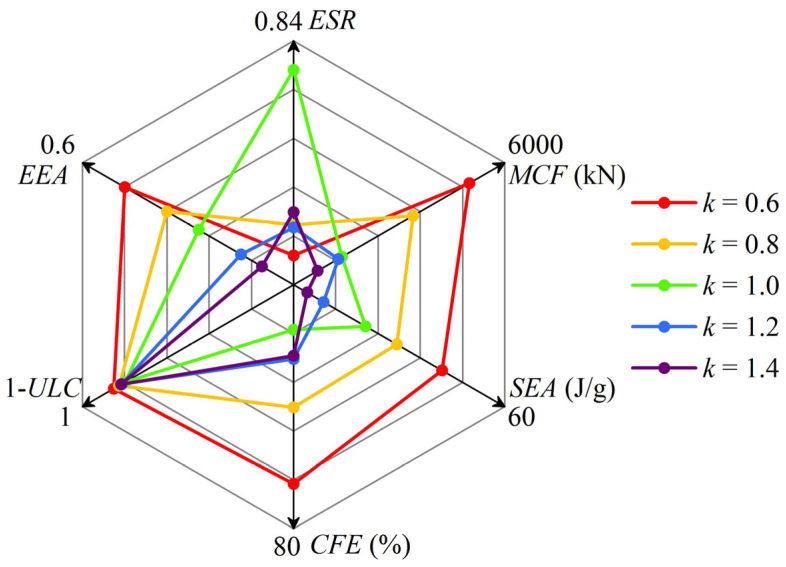
A radar diagram of the crashworthiness indicators of HPBT_C2 with different values of *k*.

To evaluate the performance of the six types of HPBT_C2 with different values of *k*, crashworthiness indicators are simultaneously presented using a radar diagram in Figure 18. It is observed that HPBT_C2, with *k* = 0.6, has the best overall performance.

## 5. Conclusions

As coal mining operations progress to greater depths, violent mining responses are inevitable. Thin-walled structures have been widely employed as energy absorbers to improve the crashworthiness of support equipment in various engineering fields. However, underground support equipment requires high-bearing capacity, which cannot be met by conventional thin-walled tubes within the limited geometrical parameters. Considering the high values of *ω* (with a large *R_T_* and a small *R_G_*) and low-cost manufacturing, this paper innovatively proposes a high-performance bi-tubular tube (HPBT) with mixed multicellular configurations. The crushing behavior and crashworthiness of the HPBT have been investigated experimentally, theoretically, and numerically. The effects of the cross-sectional shapes and wall thickness gradient distributions of the HPBT were discussed. The main conclusions are as follows:

(1) Considering the high values of *ω* (with a large *R_T_* and a small *R_G_*), low-cost manufacturing, and stable deformation modes, an innovative high-performance bi-tubular tube with mixed multicellular configurations is proposed. The theoretical model of the mean crushing force (*MCF*) is derived. The theoretical *MCF* is compared with the simulated *MCF*, and the maximum discrepancy is 6.0%, which is less than 10%. The theoretical model can provide guidance for the design of high-performance bi-tubular tubes.
(2) The specimens with conventional single-celled circular tubes were processed and subjected to axial crushing tests. The experiment and simulation results were compared with the deformation modes and force–displacement curves of specimens, fully verifying the accuracy of the finite element models and theoretical models.
(3) The design strategies of the bi-tubular structures’ mixed multicellular configurations significantly improve the values of *ω*, which enhances the crushing behaviors of the tubes. For HPBT_C2 and HPBT_S2 in particular, their *MCF*s are 4458.0 kN and 3826.5 kN, which are 28% and 34% higher than the CCT and CST, respectively. Among all the tubes studied, HPBT_C2 exhibits the highest *MCF* and *SEA*, which are 4458 kN and 46.8 J/g, respectively. The *CFE* of HPBT_C2 is 59.6%, which is 30% and 62% higher than those of the CCT and CST, respectively. The *ULC* of HPBT_C2, 0.081, is also the lowest among all the tubes studied. Considering the six indicators, HPBT_C2 shows the best crashworthy performance among all the tubes proposed.
(4) The gradient distributions (*k*) of wall thickness have a significant effect on the crashworthiness of the HPBT. At *k* = 0.4, the crushing force of the HPBT fluctuates violently without the plateau stage, making it unsuitable as an energy absorber. Moreover, *k* is equal to 0.6 and 0.8; thus, the load-bearing capacity and energy absorption of the HPBT are significantly better than those with uniform wall thickness distribution (*k* = 1). Furthermore, *k* is equal to 1.2 and 1.4; thus, the energy absorption of the HPBT decreases. Through comprehensive evaluations, the HPBT_C2 with *k* = 0.6 was found to have the best overall performance.

## Figures and Tables

**Table 1:** Geometrical parameters of high-performance bi-tubular tubes.

Type	Length of Outer Tubes *D* or *L* (mm)	Length of Inner Tubes *l* (mm)	Side of Cells *d* (mm)	Total Length of Cross-Section *B* (mm)	Thickness *T* (mm)
CCT	280.0	150	50	879.6	11.46
CST	248.1	150	50	992.5	10.16
HPBT_C1	280.0	150	50	2339.6	4.31
HPBT_C2	280.0	150	50	2139.6	4.71
HPBT_C3	280.0	150	50	2222.5	4.54
HPBT_S1	248.1	150	50	2388.9	4.22
HPBT_S2	248.1	150	50	2188.9	4.61
HPBT_S3	248.1	150	50	2271.7	4.44

**Table 2:** The mechanical properties of the material.

Density (kg/m^3^)	Young’s Modulus (GPa)	Poisson’s Ratio	Yield Stress (MPa)	Ultimate Strength (MPa)
7850	200	0.29	708.6	1063.6

**Table 3:** The *R_G_*, *R_T_, ω*, and theoretical *MCF* of all the tubes studied.

Type	*R_G_*	*R_T_*	*ω*	Theoretical *MCF* (kN)
CCT	1.00	25.13	5.01	3701.3
CST	1.13	16.76	3.63	2678.8
HPBT_C1	2.66	304.29	6.56	4346.0
HPBT_C2	2.43	257.06	6.59	4681.1
HPBT_C3	2.53	202.92	5.64	3582.6
HPBT_S1	2.72	295.92	6.33	4025.1
HPBT_S2	2.49	248.69	6.34	4027.0
HPBT_S3	2.58	194.55	5.40	3431.9

**Table 4:** Crashworthiness indicators of all the tubes studied.

Type	*ω*	*δ* (mm)	*ESR*	*EA* (kJ)	*MCF* (kN)	*SEA* (J/g)	*EEA*	*CFE* (%)	*ULC*
CCT	5.01	400.0	0.833	1398.4	3496.0	37.0	0.39	45.8	0.144
CST	3.63	440.5	0.917	1255.3	2849.8	33.2	0.35	36.7	0.231
HPBT_C1	6.56	390.5	0.814	1701.4	4357.0	45.0	0.47	57.5	0.092
HPBT_C2	6.59	397.5	0.828	1772.1	4458.0	46.8	0.49	59.6	0.081
HPBT_C3	5.64	391.0	0.815	1468.3	3755.3	38.8	0.41	50.2	0.121
HPBT_S1	6.33	395.0	0.823	1501.8	3802.1	39.7	0.40	49.9	0.098
HPBT_S2	6.34	402.0	0.838	1538.3	3826.5	40.7	0.42	50.0	0.105
HPBT_S3	5.40	401.5	0.836	1388.1	3332.7	35.4	0.37	43.7	0.142

**Table 5:** Comparison of the *MCF* of all the tubes studied between numerical simulation and the theoretical model.

Type	Simulation *MCF* (kN)	Theoretical *MCF* (kN)	Discrepancy (%)
CCT	3496.0	3701.3	−5.9
CST	2849.8	2678.8	6.0
HPBT_C1	4357.0	4346.0	0.3
HPBT_C2	4458.0	4681.1	−5.0
HPBT_C3	3755.3	3582.6	4.6
HPBT_S1	3802.1	4025.1	−5.9
HPBT_S2	3826.5	4027.0	−5.2
HPBT_S3	3332.7	3431.9	−3.0

**Table 6:** The wall thickness gradient distribution of HPBT_C2 for different values of *k*.

k	T_0_ (mm)	T_1_ (mm)	T_2_ (mm)	Mass (kg)
0.4	8.10	3.24	1.30	37.84
0.6	6.84	4.11	2.46	37.84
0.8	5.69	4.55	3.64	37.84
1.0	4.71	4.71	4.71	37.84
1.2	3.91	4.69	5.63	37.84
1.4	3.26	4.57	6.40	37.84

## Data Availability

The original contributions presented in this study are included in the article. Further inquiries can be directed to the corresponding authors.

## Abbreviations

The following abbreviations are used in this manuscript:

*A*	Net area of the tubes	*T*_0_	Wall thickness of inner multi-celled tubes
*B*	Total length of the cross-section of tubes	*T*_1_	Wall thickness of middle rib plates
*D*	Diameter of outer circular tubes	*T*_2_	Wall thickness of outer single-celled tubes
*d*	Side of cells	*U*	Ultimate tensile strength
*E*	Young’s modulus	*V*	Net volume of thin-walled tube
*E_b_*	Bending energy	*v*	Poisson’s ratio
*E_m_*	Membrane energy	*Y*	Yield stress of material
*F_C_*_1_	Working resistance of the column	*δ*	Effective stroke
*F_C_*_2_	Critical failure load of the column	*η*	Coefficient of the effective crushing distance
*F*(*z*)	Instantaneous axial crushing force	*ρ*	Density of tube wall material
*H*	Half-wavelength of lobe	*ω*	Non-dimensional parameter
*h*	Height of thin-walled tube	*σ*_0_	Flow stress of material
*k*	Coefficients of wall thickness gradient distribution	*CFE*	Crushing force efficiency
*k_d_*	Dynamic coefficient	*EA*	Energy absorption
*L*	Length of outer square tubes	*EEA*	Effectiveness of energy absorption
*l*	Length of inner tubes	*ESR*	Effective stroke ratio
*M*	Plastic bending moment per unit length	*IPCF*	Initial peak force
*m*	Mass of thin-walled tube	*MCF*	Mean crushing force
*R_G_*	Coefficient of geometric complexity	*SEA*	Specific energy absorption
*R_T_*	Coefficient of topological configuration	*ULC*	Undulation of the load-carrying capacity
*T*	Wall thickness of thin-walled tube		
